# Quality of life and depression in a cohort of female patients with chronic disease

**DOI:** 10.1186/1471-2482-12-S1-S10

**Published:** 2012-11-15

**Authors:** Fabrizio Cardin, Francesco Ambrosio, Piero Amodio, Lina Minazzato, Giancarlo Bombonato, Sami Schiff, Katiuscia Finotti, Daria Giuliani, Tonino Bianco, Claudio Terranova, Carmelo Militello, Carlo Ori

**Affiliations:** 1 Department of Surgical and Gastroenterological Sciences, Padova University Hospital, Italy, Via Giustiniani n.2, 35126 Padova, Italy; 2 Department of Medicine, Padova University Hospital, Italy, Via Giustiniani n.2, 35126 Padova, Italy; 3 Legal Medicine Unit, Department of Molecular Medicine, Padova University Hospital, Italy, Via Gabelli n.63, 35121 Padova, Italy

## Abstract

**Background:**

Differences in health-related quality of life perception in patients with chronic disease may depend on pre-existing differences in personality profile. The purpose of the study was to investigate in a cohort of female patients with chronic diseases the relationship between the Quality of Life perception and the potential presence of depressive symptoms.

**Patients and methods:**

Female patients with chronic diseases were enrolled in the study. Exclusion criteria were diagnosis of psychopathological condition, treatment with psychoactive substances.

Methodological approach was based on administration of the following test. Short Form health survey SF-36, Symptom Check List SCL-90-R, Satisfaction Profile test (SAT-P) and Beck Depression Inventory-II (BDI-II). The Pearson correlation coefficient was used to evaluate the relationship between depressive symptoms and Quality of life as assessed by psychometric test.

**Results:**

57 patients, aged 52(±3,4), responded to inclusion criteria. 57% of patients had a diagnosis of functional dyspepsia or gastro-oesophageal reflux not complicated, and the remaining 43% musculoskeletal diseases. The statistical analysis showed an inverse correlation between the variable Bodily Pain of the SF-36 and the variable Depression scales of the SCL-90-R.

In a second phase another sample of female patients was enrolled in the study. 64 patients, aged 49(±3,2), responded to inclusion criteria.

Another significant negative correlation was found between the Somatic-Affective factor of the BDI-II and the scale Physical Functioning of the SAT-P.

**Discussions:**

In female patients with chronic disease depressive symptoms resulted influenced by pain and vice versa. The treatment of depressive symptoms could improve the quality of life of patients.

## Background

Chronic diseases and their treatments, pharmacological and surgical, influence the physical and psychological health of a patient. Many studies considered this topic primarily focusing on outcome of specific surgical treatments or of treatments of oncologic pathologies [1,2]. The analysis of the influence of a chronic disease on different dimensions of quality of life (QoL) might help to identify effective treatments to improve the physical and psychological state of a patient [3].

The co-existence of depressive symptoms and a worsening of QoL during chronic diseases has been frequently described especially in old people [4]. The influence of depressive symptoms in patients with chronic conditions on QoL has been rarely studied [5,6].

In a study on QoL in Patients with Chronic Fatigue Syndrome a significant correlations between the some scale of the Symptom Check List psychopathology SCL-90-R (13) and the QoL has been found. In particular, the severity of depression was found to be the best predictor of QoL [7]. Depression also appears to be correlated to different aspects of painful symptoms such as severity, frequency and duration. Patients with pain (low back pain, headache, abdominal pain, chest pain and facial) have a probability of 2 to 5 times more to develop depressive disorders. The association between chronic pain and depressive disorders becomes stronger when increases the severity of both conditions [8].

The relationship between depression and chronic disease may have two possible explanation: 1. the chronic disease and the worsening of the quality of life are the cause of depressive symptoms; 2. a pre-morbid personality profiles determines an aggravation of pain symptoms arose in the context of chronic diseases contributing to a QoL worsening.

Based on these premises the authors considered to be interesting to explore the personality profile of patients suffering from chronic pain trying to further understand the possible impact of depressive symptoms on QoL of the patient.

## Methods

The target population was all non-institutionalised female subjects suffering from a chronic disease for at least 6 months.

The patients were recruited in the period from January-December 2011.

Exclusion criteria were as follows: male sex, presence of organic diseases and in particular of oncologic diseases, previous diagnosis of psychopathological disorders (personality disorders, mood disorders, schizophrenia and other psychotic disorders, use disorder psychoactive substances), current psychopharmacological treatment.

The methodological approach was based on administration of the following test: the Short Form health survey SF-36 [9], the Symptom Check List SCL-90-R [10], the Satisfaction Profile questionnaire (SAT-P) [^11^] and the Beck Depression Inventory-II (BDI-II) [12].

The Short Form SF-36 health survey [13] has been chosen because widely used to evaluate the QoL. It is a 36 item survey measuring health-related QoL. The following 8 subscales are constructed from the 36 items: Vitality (V), physical functioning (PF), bodily pain (BP), general health perceptions (GHP), physical role functioning (PRF), emotion role functioning (ERF), social role functioning (SRF), mental health (MH). Two summary measures known as the Physical Health Component Score (PCS) and the Mental Health Component Score (MCS) are derived. All the scales and summary measure are positively scored.

The Symptom Check List SCL-90-R, was used to obtain a subjective measurement of the state of the patient referring to the presence of symptoms in the last week. The SCL-90-R is a widely-used questionnaire that consists of 90 items, for self-report of psychological distress and multiple aspects of psychopathology, as part of the evaluation of chronic pain patients and other non-psychiatric populations [14]. From the 90 items the following subscales are derived: Somatization, Obsessive-Compulsive, Interpersonal Sensitivity, Depression, Anxiety, Hostility, Phobic Anxiety, Paranoid Ideation, Psychoticism, Sleep Disorders. The global severity index (GSI) was designed to measure overall psychological distress.

The Satisfaction Profile questionnaire (SAT-P) allowed investigating the subjective satisfaction of some aspects of daily life. The questionnaire is based on 32 items; for each item the subject is asked to provide an assessment of his level of subjective satisfaction, referring to last month. From the 32 items the following subscales are derived: Psychological Functioning, Physical Functioning, Work, Sleep/Eating/Leisure, Social Functioning.

The Beck Depression Inventory–II (BDI–II), a self-report instrument frequently used in clinical and research settings to assess depression severity was used in the Italian adaptation form. It consists of 21 groups of statements to be evaluated on a four-point scale, referring to how the subject felt in the last two weeks. From the 21 groups of statements the following subscales are derived: Somatic-Affective component and Cognitive component.

### Statistical analysis

The Pearson correlation coefficient was used to evaluate the relationship between subjective perception of their state of health (physical and psychological-emotional) and psychopathological states present in the patients. The scores obtained respectively on 8 scales of the SF-36 and on the 10 dimensions of SCL-90-R, and the scores obtained respectively on the 5 scales of the test SAT-P and 3 factors inventory BDI-II were compared.

## Results

In the first phase 57 patients, age 52(±3,4), responded to inclusion criteria. 57% of patients had a diagnosis of functional dyspepsia or gastro-oesophageal reflux not complicated, and the remaining 43% musculoskeletal diseases.

**Graph 1:**
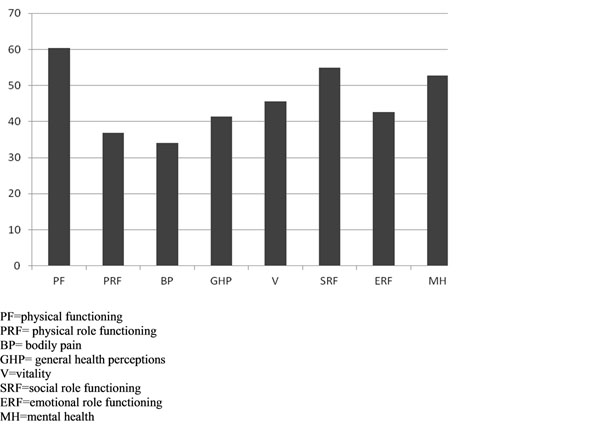
SF-36 results.

**Graph 2:**
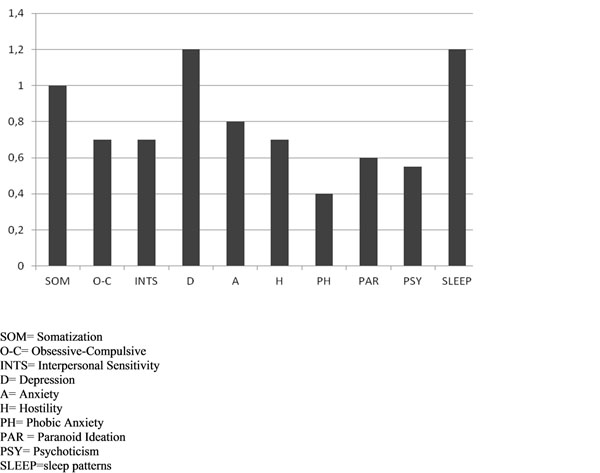
SCL-90 results.

Results of SF-36 and SCL-90 administration are graphically summarized in Graph 1 and 2.

The score on the scale of the bodily pain of the SF-36 test was 34 (± 2.1) on a scale from 0 to 100.

Physical functioning (PF), mental health (MH) and social role functioning (SRF) were the factors that recorded the highest scores (Graph 1). Bodily pain (BP) appears to be the variable most affected (Graph 1).

The test SCL-90 showed the highest score in the scale somatization (SOM), depression (D) and sleep disorders (SLEEP).

The linear correlation coefficient of Pearson reveals a significant negative correlation between the variable depression (SCL-90-R) and the variable Bodily Pain (SF-36) (Table 1). The negative correlation indicates that with an increase of the score for depression (SCL-90-R) a decrease in the score for bodily pain is observed (SF-36).

**Table 1:** Inverse correlation between the variable depression (SCL-90-R) and the variable Pain (SF-36).

R di Pearson p<.05	PF	PRF	BP	GHP	V	SRF	ERF	MH
Depression	-0.16	-0.21	-0.49*	-0.19	-0.21	-0.2	-0.14	-0.17

* p < .05

PF=physical functioning

PRF= physical role functioning

BP= bodily pain

GHP= general health perceptions

V=vitality

SRF=social role functioning

ERF=emotional role functioning

MH=mental health

In a second phase of the study another sample of female patients was enrolled in the study. 64 patients, aged 49(±3,2), with chronic disease, responded to inclusion criteria.

**Graph 3:**
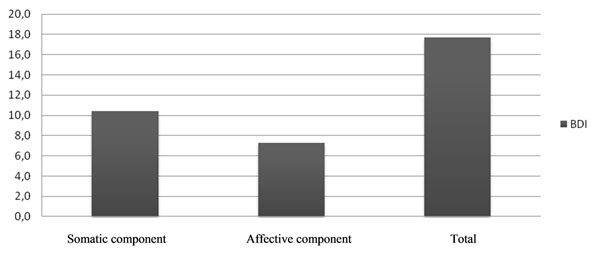
BDI results.

**Graph 4:**
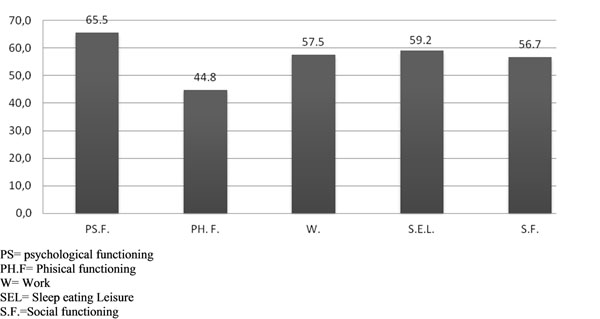
SAT-p results.

Results of BDI-II and SAT-P administration are graphically summarized in Graph 3 and 4.

At BDI somatic-affective subscale score were higher compared to cognitive subscale.

The SAT-P instead highlighted the factor of physical functioning, which stood as the only parameter under the threshold values (44.8 vs. 50). The linear correlation coefficient of Pearson reveals a significant negative correlation between the somatic-affective subscale of BDI-II and the scale physical functioning of the SAT-P questionnaire.

## Discussion

Chronic pain causes biological and psychological changes [15]. The patient with pain experiences anxiety, depression, anger, fear, and each of these emotions potentiates the action of the other, increasing the intensity of the same emotional responses [16]. The neurovegetative changes deriving from a situation of stress, represented by pain too, have a potentially negative influence on the body [17]. Depressive symptoms are probably the most frequent emotional responses to chronic pain and compromise the "functioning" of the individual, alter his ability to adapt to social life.

According to Magni et al. [18] a two-way relationship exists between chronic pain and depressive symptoms. Considering a chronic disease like musculoskeletal type disease, depressive symptoms are both predictive of the development of musculoskeletal pain and of the persistence of the latter over time. On the other hand, the presence of chronic musculoskeletal pain would be a predictor of the development, but not maintenance, of depressive symptoms.

To date, the nature of the relationship between depression and pain is still controversial.

The analysis of the relationship depression-QoL in chronic disease could have relevant consequences from the therapeutic point of view; depressive symptoms could influence the perception of physical pain and the pharmacological treatment [19] consequently could allow improving the QoL. Recently Lerman et al. [20] distinguished pain from depression in patients with chronic disease. In particular they differentiated the somatic aspects of depression from the negative emotions related to the chronic disease.

In our study the SCL-90-R administration allowed detecting the presence of depressive symptoms subdivided into emotional-affective, cognitive and somatic components by means of different 13 items. Depressive symptoms were related to different aspects explored by the SF-36 and in particular to the Physical Health Component Score (specifically to role physical, bodily pain, general health). At the same time it was possible by means of the SF-36 questionnaire administration to evaluate the variable bodily pain using two items.

The results of BDI and SAT-p are consistent with results of previous test.

Considered the causality relation between depression and physical pain, we hypothesized that the depression experienced by patients with chronic pain is similar to that one described by Lalli [21]; he described a form of depression, called "masked depression" that does not have the typical psychic symptoms of depression (sadness, pessimism, psychic pain) but only somatic symptoms (including apathy, insomnia, fatigue, loss of libido, dyspeptic disorders), some of which investigated by the depression dimension of the SCL-90-R. Latent depression in the subject would emerge with a single symptom, represented by the "psychogenic pain" that may be present in various areas of the body in the form of physical pain. This hypothesis seems to strengthen the reflections made by Magni [18] about the nature of the relationship between the two variables.

The existence of an effect of depressive symptoms on the development and persistence over time of chronic pain may be motivated by assuming that when the pain occurs in the presence of depressive symptoms it may be a somatic manifestation of an underlying psychological disorder.

In the light of these considerations, it would be interesting to further investigate the nature of depression, considering whether there are gender-specific about the relationship between the subjective perception of their state of health (physical and psychological-emotional) and the evaluation of potential psychopathological states extending the study compared to males.

**Table 2:** Inverse correlation between the somatic subscale of BDI-II and the scale physical functioning of the SAT-P questionnaire.

R Pearson p<.5	Somatic component	Affective component	BDI scoring
Phisical functioning	-0.76*	-0.21	-0.29

* p < .05

## Competing interest

The authors declare that they have not competing interest.

## Authors’ contributions

FC contributed to the recruitment of the patients, to literature review, to the drafting and reviewing of the paper. FA has made substantial contributions to acquisition analysis and interpretation of data. PA, LM and GB have made substantial contributions to conception and design of the paper, and have been involved in drafting the manuscript and revising it critically for important intellectual content. SS, KF, DG have been involved in collecting and interpreting data, in drafting the manuscript. TB has made statistical analysis and interpretation of the data, to the discussion and to the writing of the paper. CT gave his contribution to the analysis and interpretation of the data, to the discussion and to the writing of the paper. CM gave his contribution to the interpretation of the data and to the review and writing of the paper. All the authors read and approved the final manuscript. CO, has made substantial contributions to the analysis and interpretation of the data and to the review and writing of the paper.
